# Chaos in heterogeneous neural networks: I. The critical transition point

**DOI:** 10.1186/1471-2202-15-S1-O20

**Published:** 2014-07-21

**Authors:** Johnatan Aljadeff, Merav Stern, Tatyana O Sharpee

**Affiliations:** 1 Dept. of Physics and Center for Theoretical Biological Physics, University of California San Diego; Computational Neurobiology Laboratory, The Salk Institute for Biological Studies, La Jolla, CA, 92037, USA; 2 Dept. of Neuroscience, Columbia University, New York, NY, 10027, USA; Interdisciplinary Center for Neural Computation, Hebrew University. Jerusalem, 91905, Israel

## 

There is accumulating evidence that biological neural networks posses optimal computational capacity when they are at or near a critical point in which the network transitions to a chaotic regime. We derive a formula for the critical point of a general heterogeneous neural network. This formula relates the structure of the network to its critical point. The heterogeneity of the network may describe the spatial structure, a multiplicity of cell types or any selective connectivity rules.

To define the network we divide the *N* neurons into *D* groups such that *∑_d_*_=_*_1...D_N_d_*=*N*. The synaptic weight between neurons *i*,*j* (the connectivity matrix element *J_ij_*) is drawn from a centered distribution with standard deviation summarized in a *D*×*D* rule matrix *N^-1/2^G_c_*_(_*_i_*_)_*_d_*_(_*_j_*_)_(insets to **A, ***c*(*i*) is the type index of neuron *i*). The network obeys the standard rate dynamics (*d/dt*)*x_i_*=*- x_i_*+*∑_j_*_=_*_1...N_J_ij_* tanh*x_j_*.

The global behavior of the network changes from a single fixed point to chaos when *r*=*1*, *r* being the radius of the circle that bounds the spectrum of the connectivity matrix (panel **A**). We derived a formula, in terms of the matrix *G* and the vector *N_d_*, for *r* that can also be thought of as an effective gain[1]: it is the square root of the maximal eigenvalue of a *D*×*D* matrix *M* whose *c*,*d* element is *M_cd_*= *N^-1^N_c_*(*G_cd_*)*^2^*.

**Figure 1:**
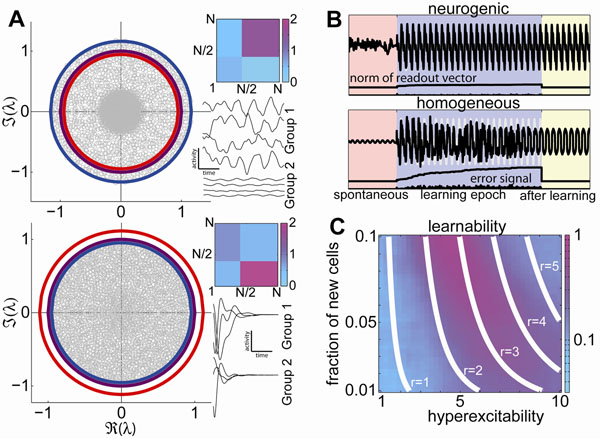
**(A)** Example spectra of connectivity matrices (gray) with *r*>*1* (top, indicated in blue and purple respectively) and *r*<*1* (bottom). The average synaptic gain (red) does not give the correct boundary of the spectrum and would predict opposite behavior. The matrix *G_c_*_(_*_i_*_)_*_d_*_(_*_j_*_)_ is indicated by the color plots (top), and activity of representative neurons from the two groups (bottom) of each example. **(B)** The activity of a readout unit during spontaneous activity, a FORCE learning epoch, and post learning for neurogenic and homogeneous subcritical networks. The neurogenic network quickly matches the target signal (gray) and robustly reproduces it. **(C)** The learnability of an ensemble of neurogenic networks as a function of the hyperexcitability and new neuron fraction coincides with contour lines of *r* (white).

We use our understanding of the general heterogeneous dynamical system to a network with a large fraction of cells in the subcritical regime, and a small fraction of supercritical neurons. This can be thought of as a model of a network where adult neurogenesis occurs, where a small fraction of hyperexcitable neurons are continuously integrated. Using a supervised learning algorithm (FORCE, [2]) we show that *r* is as a good coordinate to describe the network's “learnability” (Figure 1 panels B,C). Learning is optimal for values of *r* similar to those found in a homogenous network. Our results suggest that the new neurons can allow the network to be poised at criticality with no global changes to connectivity, and that their specific roles are context dependent, in contrast to previous hypotheses.
